# Spatiotemporally transcriptomic analyses of floral buds reveal the high-resolution landscape of flower development and dormancy regulation in peach

**DOI:** 10.1093/hr/uhaf029

**Published:** 2025-02-11

**Authors:** Ya-lin Zhao, Yong Li, Dan-dan Guo, Xue-jia Chen, Ke Cao, Jin-long Wu, Wei-chao Fang, Chang-wen Chen, Xin-wei Wang, Li-rong Wang

**Affiliations:** Zhengzhou Fruit Research Institute, Chinese Academy of Agricultural Sciences, Zhengzhou, Henan 450009, China; College of Horticulture and Landscape Architecture, Henan Institute of Science and Technology, Xinxiang, Henan 453003, China; Zhengzhou Fruit Research Institute, Chinese Academy of Agricultural Sciences, Zhengzhou, Henan 450009, China; Institute of Western Agriculture, Chinese Academy of Agricultural Sciences, Changji, Xinjiang 831100, China; Zhengzhou Fruit Research Institute, Chinese Academy of Agricultural Sciences, Zhengzhou, Henan 450009, China; Zhengzhou Fruit Research Institute, Chinese Academy of Agricultural Sciences, Zhengzhou, Henan 450009, China; Zhengzhou Fruit Research Institute, Chinese Academy of Agricultural Sciences, Zhengzhou, Henan 450009, China; Zhengzhou Fruit Research Institute, Chinese Academy of Agricultural Sciences, Zhengzhou, Henan 450009, China; Zhengzhou Fruit Research Institute, Chinese Academy of Agricultural Sciences, Zhengzhou, Henan 450009, China; Zhengzhou Fruit Research Institute, Chinese Academy of Agricultural Sciences, Zhengzhou, Henan 450009, China; Zhengzhou Fruit Research Institute, Chinese Academy of Agricultural Sciences, Zhengzhou, Henan 450009, China; Institute of Western Agriculture, Chinese Academy of Agricultural Sciences, Changji, Xinjiang 831100, China; Zhengzhou Fruit Research Institute, Chinese Academy of Agricultural Sciences, Zhengzhou, Henan 450009, China; Institute of Western Agriculture, Chinese Academy of Agricultural Sciences, Changji, Xinjiang 831100, China

## Abstract

The spatiotemporal transcriptome dataset reported here provides the peach flower bud’s gene expression atlas at spatiotemporal resolution level using the 10x Genomics Visium platform. This dataset can be used to define transcript accumulation for any interesting genes across several flower bud cells. It was generated using three peach flower bud samples during the activity–dormancy period, providing valuable insight into gene expression profiling and developmental stages under different environmental contexts or conditions. Importantly, we found that different cell types are related to specific regulatory programs, including signal transduction, environment and stress responses, and flower development. Our research provides insight into the transcriptomic landscape of the key cell types for flower buds and opens new avenues to study cell-type specification, function, and differentiation in Rosaceae fruit trees. A series of pivotal genes (e.g. *AMS*, *MS188*, *MS1*) for flower bud development were identified. These results provide a valuable reference for the activity–dormancy transition in perennial deciduous fruit trees.

## Introduction

Plant bud dormancy is an adaptive characteristic in response to harsh environment cues, and is conducive to the preservation of flower buds, enabling them to survive the cold winter and have better recovery of growth, flowering, and fruit in the following year. The release time of plant bud dormancy is highly dependent on changes in external environmental conditions, and seasonal variations in bud break and flowering time have been reported. It is worth noting that the germination and flowering dates of some fruit tree species, such as peach, apple, and apricot, are advanced in the warmer spring months [1, 2]. The advancement of bud break increases the risk of late frost damage in the northern hemisphere. Moreover, insufficient cold accumulation in winter may lead to incomplete dormancy release, resulting in a decline in yield [3]. Bud dormancy release of peach has a very strict requirement of low temperature [4]. Therefore, there is an urgent need to better understand bud responses to environmental signals to better address fruit loss and predict future production changes.

Accumulation of chilling in winter as a requirement for flowering, fruit yield, and quality has been known for over two centuries [5]. Several models have been used to quantify chilling accumulation and estimate dormancy release based on hourly temperature data, such as a dynamic model, the Utah model and the Weinberger model [6–9]. However, due to the lack of detailed understanding of dormancy regulation, the lack of standardization has limited its applicability to other regions with different climatic conditions. Pollen meiosis could act as a key stage for the activity–dormancy transition. Several studies have focused on the relationship between meiosis and callose deposition during pollen formation and dormancy release [10, 11]. Once the chilling requirement (CR) is complete, meiosis follows closely and is highly related to dormancy release [11]. Male meiosis is completed within 1 week, followed by a change in anther color from green to yellowish [12]. Gametophyte development is dependent on nutrients and cell wall material in the sporophyte cells, and growth hormones produced by flavin monooxygenases (YUCs) are essential for the early stages of pollen development [13]. Sporopollenin biosynthetic proteins, a major component of the pollen outer wall, are specifically expressed in the tapetum and then secreted into anther locules. CYP704B is responsible for sporopollenin synthesis [14]. Mutations of several related genes that encode transcription factors (TFs) cause the additional vacuolation and the irregular degradation of the tapetum. These TFs cooperatively form the tapetal regulation pathway (DYT1-TDF1-AMS-MS188-MS1) and precisely control its development [15]. Tapetal cells contain numerous mitochondria, which are beneficial for the delivery of nutrients to microspores located in pollen sacs [16, 17]. It has been shown that overwintering at the meiosis stage of microspores is associated with the largest percentage of abnormality, while pre- or post-meiotic stages appear to be more stable [18]. The sporogenous stage also appears to be cold-tolerant due to its meristematic nature. The exine layer of pollen most likely consists of sporopollenin, which is derived from long-chain fatty acids, oxygenated aromatic rings, and phenylpropionic acids [19]. However, the relationship between these genes and the flower bud activity–dormancy transition has not been expounded in detail in peach.

The activity–dormancy transition is a phenotype influenced by complex factors. MADS-box genes widely affected dormancy control, flower development, and identity determination of organs [20–22]. Six *Dormancy Associated MADS-box* (*DAM*) genes exist in the form of tandem replication to control dormancy in peach [23]. Members of the plant-specific *WUSCHEL* (*WUS*)-related homeobox (*WOX*) genes play crucial developmental roles through hormone pathways in flower bud development [24]. In addition, ABA inhibits cell proliferation and shoot growth and can induce dormancy through ABA catabolism, biosynthesis, and promotion of signaling in the terminal bud set [25, 26]. GA may play the opposite role to ABA during the dormancy–activity transition process [27, 28]. However, the spatiotemporal expression of these genes has not been studied in detail.

The use of single-cell RNA sequencing (scRNA-seq) led to a significant breakthrough in *Arabidopsis thaliana* root cells, where it identified distinct subpopulations and rare cell types and provided a first-generation gene expression atlas at single-cell resolution [29], and this method has demonstrated feasibility and utility in plants. Since then, scRNA-seq has been widely used in different plant species, such as rice [30, 31], maize [32], tomato [33], and *Populus* [34]. These results identified many new cell types and marker genes, laying the foundation for follow-up functional research on each tissue function response to different environments. Even though single-cell sequencing is so widely used, it does not provide spatial and location information on the cells [35]. Recent advances in spatial transcriptomics sequencing (ST-seq) have addressed this question. Here, we performed spatiotemporal transcriptome sequencing of peach flower buds before anthesis, which provides a comprehensive understanding of the functions of different tissue types during flower bud development alongside changes in environmental cues. We have used ST-seq to profile the atlas of peach flower bud cells and constructed a genome-scale gene expression resource covering the dormancy–activity transition during the early reproductive development phase. The differentiation trajectories of flower organs were reconstructed, discrete cell types and groups of regulators in the highly heterogeneous young inflorescence were identified, and the anther development-related genes were validated by *in situ* hybridization. These results provide a crucial and elaborate reference for activity–dormancy research.

## Results

### Cell-type recognition in flower bud based on spatial transcriptome sequencing

**Figure 1:**
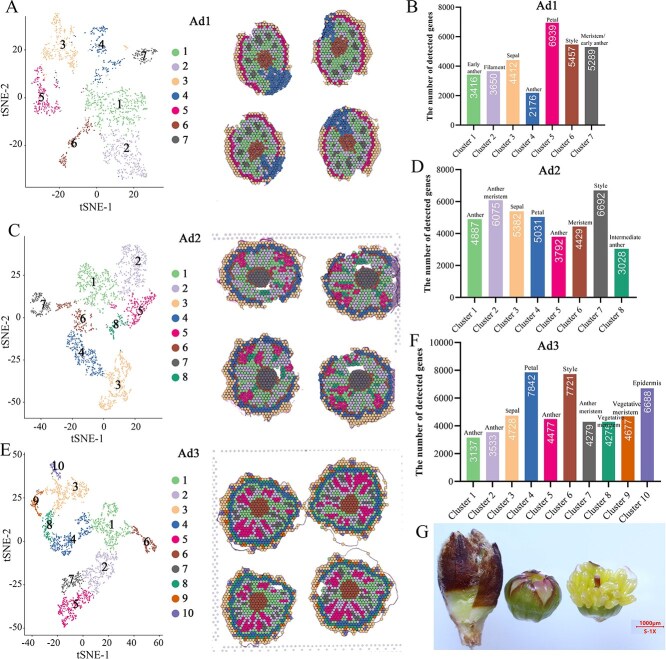
t-SNE cluster analysis and cell type identification of flower bud samples at three different development stages. **A** Clusters were obtained of the first sample by unsupervised clustering using Seurat and visualized based on t-SNE algorithms. The figure on the right of panel **A** shows the specific distribution of different groups in the sample space slice position, and the color corresponds to **A**. **C** and **E** represent the second flower and third sample, respectively. The figures on the right of panel **C** and **E** show the specific distributions of different groups in the sample space slice position, and the color corresponds to **C** and **E**, respectively. **B** Number of detected genes in each cluster of sample Ad1. **D** corresponds to the Ad2 sample and **F** represents the Ad3 sample. **G** The development state of Ad3, its anther color turning yellowish.

In order to provide a spatiotemporal cellular map of the buds to gain a full understanding of the complexity, we performed ST-seq using flower bud transverse planes of three samples, spanning dormancy to dormancy release stages, and covering the stage from the existence of the stamen to nearly mature pollen gain formation on a 10x Visium platform. The first sample (Ad1) exhibited the earlier development stage of the floral bud, consisting of distinguishable sepal, petal, the earlier stamen and pistil. Spatial transcriptome sequencing of the samples yielded 1836 effective beads with 23 629 effective genes in all clusters, of which 3406 genes (Supplementary Data Table S1) showed significant spatial differences between different clusters (Fig. 1A). Application of t-distributed stochastic neighborhood embedding (t-SNE) based on the expression data and HE staining classified these spots into seven clusters of stamens and filament (clusters 1, 2, 4, 7), sepal (cluster 3), petal (cluster 5), and style (cluster 6) (Fig. 1A). These results revealed that a high degree of cell heterogeneity exists in early flower bud development and that the spatiotemporal transcriptome dataset can facilitate the identification of discrete cell types and developmental stages in peach flower buds. Detailed exploration of the spatial expression properties of the known and key genes, such as specific or preferential expression in one or more tissues, has been a great support for cell classification, e.g. an HB-WOX TF (*Prupe.5G232600*) in cluster 6 (Supplementary Data Table S2), which showed significant expression in the style and is a homolog of *OsWOX*, important for flower meristem activity in rice inflorescence meristems [31]. An LTP family protein gene, *Prupe.5G220400*, was specifically expressed in clusters 1 and 7 (Supplementary Data Table S2), which played a key role in anther development [36]. The highest number of expressed genes was found in sepal and petal tissues and accounted for 36.2% of the total number of expressed genes in all floral organs (Fig. 1B).

The second sample analyzed (Ad2) covered an entire bud at dormancy release stage, having 2348 valid spots and 39 047 valid genes including repeated genes among the different clusters, of which 39 021 showed spatial variation in expression (*P* < 0.05) (Supplementary Data Table S3). One eight-group clustering of the plane yielded cell types representing relatively matured floral buds including stamens and filament (clusters 1, 2, 5, 6, 8), sepals (cluster 3), petal (cluster 4), and style (cluster 7) (Fig. 1C). The flower buds became more expansive; based on the global expression similarity of the spatially variant genes, all tissues were well clustered and in high agreement with the observations of the corresponding histological examinations (Supplementary Data Fig. S1). The expressed gene number in sepal and petal tissues accounted for 26.4% of the total expressed gene number in whole tissues (Fig. 1D). This showed that the development rate of Ad2 reproductive tissue is relatively faster than that of Ad1 in the dormancy release state.

The third flower bud analyzed (Ad3) at eco-dormancy stage covered 2677 spots with 51 357 expressed genes including repeated genes in all the clusters. Ten clusters were obtained by t-SNE dimension reduction analysis, suggesting the internal differentiation of the flower bud was accelerated when the flower bud reached the dormancy release stage (Fig. 1E). Highly consistent with histological observations (Supplementary Data Fig. S1), reproductive tissues had the most diversity, occupying 8 of the total 10 clusters, far more than those in relatively mature organs like the surrounding sepals and petals with one cluster, respectively. Analysis of the number of gene expressions and clusters showed that the internal reproductive tissues continued to develop (Fig. 1F), and several TF genes played key roles in flower organ identification (Supplementary Data Table S4). The third sample was composed of a bud at a late morphogenesis stage before anthesis; the color of the anther turned from green to yellowish (Fig. 1G). To be clear, the outer scales were removed before tissue fixation and embedding to fit in the slide.

In short, 1836, 2348, and 2677 valid spots were obtained in the three samples, respectively. The mean reads per spot were 159 324, 168 278, and 191 824 and the median genes per spot were 6649, 8838, and 8422, respectively (Supplementary Data Table S1). After linear dimensional reduction, t-SNE tools and the uniform manifold approximation and projection (UMAP) algorithm were used to visualize and explore the spatial transcriptome (ST) datasets [30]. Unsupervised analyses revealed 7, 8, and 10 major clusters in the flower bud based on UMAP or t-SNE alongside the development process, respectively (Supplementary Data Fig. S2).

### Trajectory analysis reveals the sustained inward differentiation progression of the flower bud

**Figure 2:**
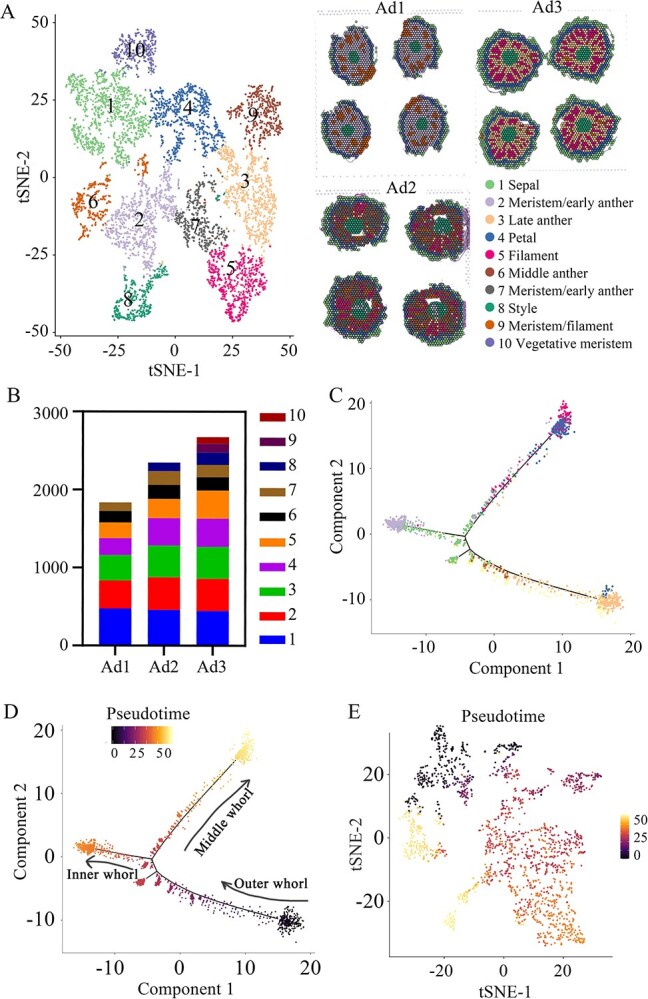
Reconstruction of cell identity and differentiation trajectory using all three peach flower bud samples. **A** Clusters were obtained of the whole of three samples by unsupervised clustering using Seurat and visualized based on the t-SNE algorithm using 6861 spots. The cell type of each cluster was identified. In the right panel is the clustering information of AD1, AD2, and AD3 samples. **B** Spot number in each cell type. **C**–**E** Pseudotime trajectory of cell-type profiles for seven clusters of Ad1. The black arrows represent the differentiation direction.

To fully investigate the cell heterogeneity during flower bud development, the three samples were combined for further analysis. The result showed that 10 clusters in total were separated, which is consistent with the result of Ad3 clustering based on dimensionality reduction clustering at 0.4 resolution (Figs 1E and 2A), demonstrating the continuity of cell developmental processes. The cluster number of the outer whorls remained unchanged, and the increased cluster number appeared in the stamen (Fig. 2A). This result showed that completion of the development of the external tissue is earlier than that of the inner tissue, demonstrating the development trajectory of the flower bud with the ‘outside-in’ dynamic development model (Fig. 2A). Accordingly, the number of external spots remained essentially constant, while the number of spots in inner tissues increased (Fig. 2B). To examine the trajectory of these developmentally related cell types, pseudotime analysis was applied to the clusters representing these cell types [37]. Sample Ad1, taken as an example here, also provides evidence of outside-in developmental trajectories (Fig. 2C–E). An individual spot was projected onto two ends of the pseudotime backbone, representing two distinct final states (Fig. 2C and D); the development state was also revealed by the expression state in pseudotime (Fig. 2E). Additionally, anthers/pollen have specific gene expression profiles [38], which may contribute to the identification of clusters.

A Pearson correlation coefficient of >0.9 between two adjacent periods was calculated, and the value between Ad1 and Ad2 was slightly larger than between Ad2 and Ad3 (Supplementary Data Fig. S3). Systematic cluster analysis also supported this point (Supplementary Data Fig. S4). In addition, from the perspective of valid spots from the three samples, the number of expressed genes in peach flower buds did not stop increasing, even when they were in the dormant stage [39], reproductive tissues accounted for the majority of flower organ types, unlike apple and pear buds, which remain largely unchanged during dormancy and rapidly grow in late eco-dormancy [40, 41]. These results showed that cells in peach flower buds are still differentiating even under low temperatures, possibly as a result of mitosis or meiosis.

### Gene Ontology and Kyoto Encyclopedia of Genes and Genomes analyses of all clusters

**Figure 3:**
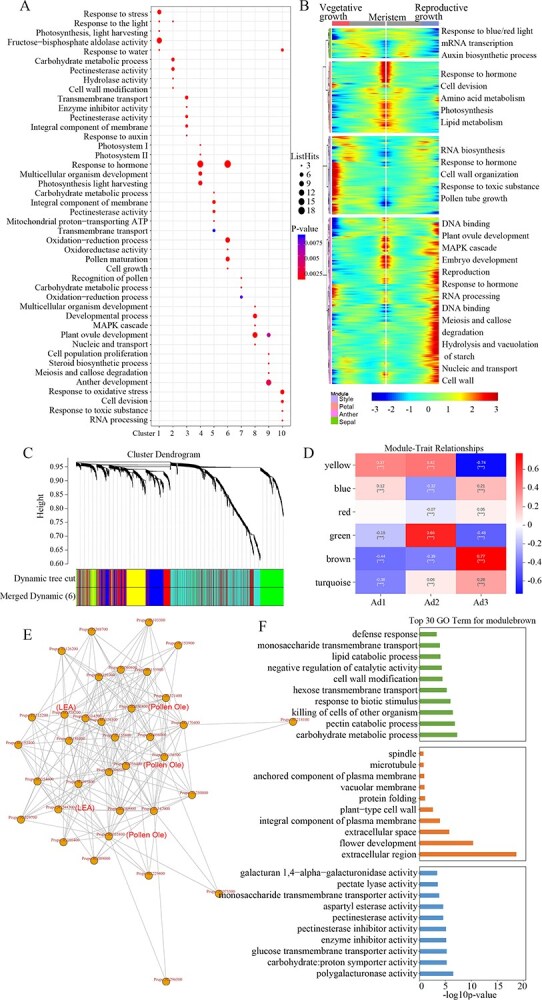
Gene ontology (GO) analysis of all 10 clusters and anther development regulators identified using weighted gene co-expression network analysis (WGCNA). **A** GO analysis for the top five terms in all 10 clusters. Significant enrichment is represented by the *P*-value and the amount of enriched genes is represented by circle size. **B** Heat map showing expression of DEGs in flower buds over branch point 1 and point 2 of pseudotime. The white line in the middle represents the beginning of pseudotime (meristem); the two sides represent the ends of pseudotime down the two lineages (left panel, vegetative tissues; right panel, reproductive tissues). **C** Hierarchical clustering tree structured based on WGCNA. Each leaf in the tree represents one gene. **D** Correlation heat map between different modules and three samples. With *P* ≤ 0.05 as the threshold value, the module related to anther development was screened. **E** Gene interaction network analysis in the brown modules. Lines in the network indicate the degree of connection between genes. The more lines there are, the greater the number of connections. **F** GO functional enrichment analysis of the top 30 terms for genes in brown modules.

To further provide the basis for cell typing in all flower bud samples, we combined the samples of all three periods, and the differentially expressed genes (DEGs) were identified in each cluster for enrichment analysis. Correspondingly, in Gene Ontology (GO) enrichment analysis we observed that three clusters [19, 42, 43] were significantly enriched in stress response (oxidative/toxic substance), light (photosystem I/II), and hormone, cell division, multicellular organism development and fructose-bisphosphate aldolase activity (Fig. 3A), all of which are involved in sepal and petal formation under environmental stress. Anther development and maturation, plant ovule development, pectinesterase activity, cell wall modification, meiosis, and callose degradation were enriched in seven clusters [3, 18, 23, 24, 39, 44, 45] for the reproductive cells (Fig. 3A), and played key roles in pistil and stamen development (Fig. 3A). The DEGs were correlated with the estimated pseudotime of these clusters, showing either increasing expression levels with pseudotime or decreasing ones (Fig. 3B). Preferential genes in sepal and petal tissue were enriched in categories of mRNA transcription, auxin biosynthetic and lipid metabolism processes (Fig. 3B), and in GO terms of response to stimulus (Fig. 3A), indicating the activities of photosynthesis in differentiated cell types. On the contrary, genes preferential in stamen and carpels were enriched in meiosis and callose deposition, embryo development, reproduction, plant ovule, or anther development. For example, *PpCALS1/2* (callose synthase enzyme), positively regulated by *PpDAM6* in our previous study [46], clearly revealed the process of early callose biosynthesis of reproductive tissues (Supplementary Data Fig. S5).

In Kyoto Encyclopedia of Genes and Genomes (KEGG) analysis, clusters 1 and 4 were significantly enriched in plant hormone signal transduction and photosynthesis, and these enriched genes encode all necessary enzymes for carbohydrate and amino acid metabolism process (Supplementary Data Fig. S6), indicating rapid sepal and petal growth in clusters 1 and 4. Cluster 10 was significantly enriched in alternative splicing and cyanoamino acid metabolism, indicating DEGs involved in defense signaling and the synthesis of defense-related metabolites (Supplementary Data Fig. S6). Seven clusters of reproductive tissue [3, 18, 23, 24, 39, 44, 45] were significantly enriched in plant hormone signal transduction, MAPK signaling pathway, TCA cycle, ABC transporters, linoleic acid metabolism, fatty acid biosynthesis, oxygenated aromatic rings and phenylpropionic acids (Supplementary Data Fig. S6). These results showed that these processes played a key part in exine layer formation of pollen.

To find the anther development-related module, 2974 genes from 6861 spots (SD ≤ 1e−10) were selected for weighted gene co-expression network analysis (WGCNA). After merging modules with high similarity (80%), six modules with different color markers were finally identified (Fig. 3C and D). Then, the correlation between the characterized genes and flower development was calculated. The Pearson correlation coefficient of one of the modules (brown color) was >0.7, suggesting that it might function in controlling anther development (Fig. 3D). The correlation values showed a big difference between Ad2 and Ad3 samples, suggesting their variety in development degrees (Fig. 3D). The top 50 genes with the highest connectivity in the brown module were analyzed to show the relationship between these genes. Three pollen Ole genes and two LEA genes were identified (Fig. 3E), which suggested these genes play key roles in the activity–dormancy cycle [47, 48]. The GO enrichment analysis of the brown module revealed over-represented GO terms related to carbohydrate metabolic process, cell wall modification, protein folding, and responses to stress stimulus (Fig. 3F).

### Spatiotemporal gene expression dynamics during cold development of flower buds

**Figure 4:**
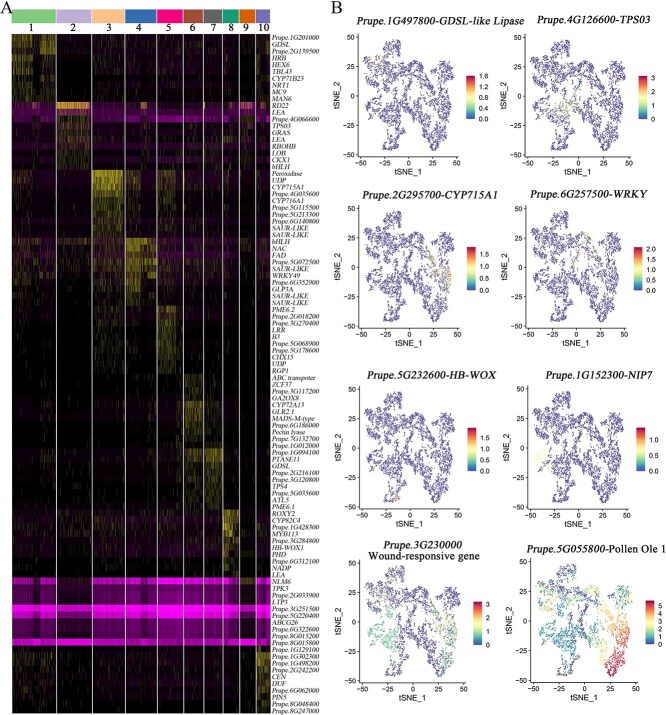
Identification of key genes in all 10 clusters and marker gene expression on t-SNE projection plots during cold exposure stages. **A** Heat map analysis of expression levels of representative marker genes in each cluster. **B** Feature plots show expression levels of representative marker genes in key cell types.

To explore the function of potential regulators alongside dormant bud development, we analyzed the differential gene expression profiles of different cell clusters and identified key regulatory genes with high expression in their corresponding cell clusters (Fig. 4A and B). A gene of the NAC family was identified in cluster 4, *Prupe.1G587100,* which constitutes one of the largest groups of plant-specific TFs and is known to play essential roles in abiotic stresses such as dehydration and cold stress, [49] (Fig. 4A). We identified an SBP-box gene, *Prupe.2G134900*, a homolog of *SPL8*, important for sepal and stamen development in *Arabidopsis* [50], which was preferentially expressed in clusters 2, 4, 8, and 9. This gene may function downstream of AGAMOUS (AG) (C-class), which is the regulator of sporogenesis [51].

Consistent with the report that the *SAUR* (*SMALL AUXIN UP RNA*) gene is highly expressed in reproductive cells and is required for efficient pollen tube elongation and subsequent fertilization, and possibly regulates the development of flower organs and cell division [52, 53], our results show that *SAUR-LIKE* genes are highly expressed in anther and petal, indicating the diversity of gene function (Fig. 4A). Some of these marker genes could potentially be involved in regulating the development of anther cells, e.g. MADS-box and SCAP1 [42, 46]. The bHLH TF played a key role in stress tolerance and regulation of flower development [54, 55]; we screened two highly and differently expressed bHLH TFs in different cell types (Fig. 4A). One of the *Prupe.1G006600* genes was preferentially expressed in cluster 4 and might play key roles in diverse biological processes, including photomorphogenesis and biotic and abiotic stress responses [54] (Fig. 4A). Additionally, representative marker genes from different clusters were also identified for a more intuitive presentation (Fig. 4B). For example, a GDSL lipase is required for anther development [56]; however, the gene is highly expressed in sepal cells. *PpTPS03* may interact with *PpABI5* to enhance abiotic stress by indirectly participating in the ABA signaling pathway [57]. The cytochrome P450 gene *CYP715A1* (*Prupe.2G295700*) was identified (Fig. 4B), and the result revealed that ABA content played a crucial role in CR-mediated flower bud dormancy process [26]. The two TFs *LRR* and *NIP7* are potentially involved in anther development and pollen exine cell wall formation [58, 59] (Fig. 4A and B).

In our study, it is worth noting that two different co-expressed genes were screened in all three development stages using the top 10 DEGs from each cluster, and were preferentially and significantly expressed in the style. One of these is an HB-WOX TF (*Prupe.5G232600*), a homologous gene of *OsWOX*, a marker gene of the style in rice, regulating flower meristem activity and inflorescence meristems [31], and the HB-WOX gene, preferentially expressed in the style of all samples (Fig. 4A and B). Another one is a 2-methylene-furan-3-one reductase isoform gene (*Prupe.3G284800*), which may be related to the biochemical reaction of style development (Supplementary Data Tables S5–S7). Peculiarly, the two highly expressed DEGs, wound-response gene (*Prupe.3G230000*) and Pollen Ole e 1 (*Prupe.5G055800*), were screened in most clusters. Interestingly, the former displayed high expression levels in the style and the latter showed high expression in reproductive cells (Fig. 4B).

### Spatial expression of MADS-box genes revealed floral organ identity determination under cold acclimation

**Figure 5:**
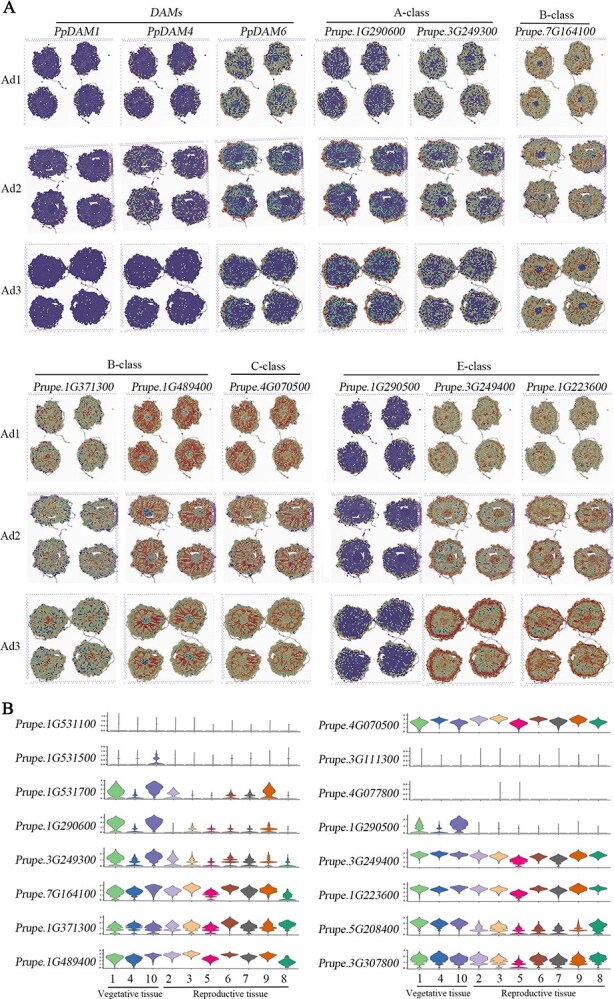
Spatial expression distributions of MADS-box genes involved in floral organ identity determination. **A** Expression trend analysis of MADS-box genes in different dormancy periods. **B** Violin plots corresponding to the upper panel show quantitative expression levels of representative marker genes in different tissues.

Only three genes (*DAM1*, *DAM4*, *DAM*6) out of all six annotated *DAM* genes (*PpDAM1–6*) were detected in the dataset. This may be caused by gene function redundancy, consistent with our earlier results on the peach flower bud transcriptome [60]. Their expression levels showed a downward trend alongside dormancy release [20, 22], and were closely related to dormancy control and the flower bud development process. A previous study revealed that the three *DAM* genes had a successively decreased expression level from carpel to petal and stamen [61]. The *PpDAM1* gene showed a slightly low expression level, contrary to previously reported gene function results [62]; however, the overall expression trend of the other two genes (*PpDAM4* and *PpDAM6*) was downward in reproductive organs when compared with vegetative tissue, suggesting its correlation with germ cell development control alongside the dormancy release process (Fig. 5A and B, Supplementary Data Table S8), consistent with our earlier study [46].

In *Arabidopsis*, *Oryza sativa*, and many other eudicots, the A-function, essential for identity determination of sepal and petal, is implemented by multiple duplicated A-class genes (AP1). Similarly, AP1-like genes *Prupe.1G290600* and *Prupe.3G249300* were preferentially expressed in sepal or widely expressed in anther during the dormancy process, similar to *MdMADS12*, an AP1-like A-function gene, mainly expressed in vegetative organs in apple in a previous study [63] (Fig. 5A and B, Supplementary Data Table S8).

The functions of B- and C-class genes are highly conserved in angiosperms, and they are essential for both reproductive and vegetative identity. Previous studies on peach have shown that both PI- and AP3-like genes (B-class) are fully expressed in petals [45]. Consistently, the two AP3-like genes (*Prupe.7G164100* and *Prupe.1G371300*) of peach presented preferential and different expression patterns revealed by spatial transcriptomic data. Among them, although slightly expressed in the sepals of Ad1, the main expression of *Prupe.7G164100* was in anther rather than in petal. Also, *Prupe.1G371300* showed higher expression in stamens and carpels and was expressed only in petals of the third sample, consistent with the *PpAP3* function of a previous study in peach [45] and *MdMADS13*, an AP3-like B-function MADS-box gene, mainly expressed in petals and stamens in apple [63] (Fig. 5A and B). A PI gene (*Prupe.1G489400*) was preferentially expressed in the stamen alongside the development process except for its expression in the petal of the Ad3 sample (Fig. 5A and B).

We found four AG-like (C-class) genes in the peach genome; the same bead was also highlighted in the spatial expression profile of *Prupe.4G070500* (AG-like, C-class), providing further evidence of the organogenesis of the anther. Meanwhile, another gene *Prupe.3G111300* was only expressed in the pistil in the last sample, suggesting that it plays a crucial role at a specific stage of reproductive development. However, the remaining two genes (*Prupe.4G077800* and *Prupe.8G152000*) had no spatial expression detected in all samples (Fig. 5A and B).

It was well known that SEP genes (E-class) are involved in the development of reproductive organs. However, the *Prupe.1G290500* gene was mainly expressed in outer whorls in all samples, and its expression trended spread to anther only in the third sample, which revealed preferential and different expression patterns based on the spatial transcriptomic data. The expression pattern of another gene (*Prupe.1G223600*) was opposite to the last one, and distinctly appeared in the stamen and pistil in all samples. However, the *Prupe.3G249400* gene, in addition to containing the last gene expression region, was also active in the outer whorl just like the first one, showing its correlation with the natural development of both reproductive and vegetative tissues (Fig. 5A and B).

### Reproductive meristem development responds to cold exposure and phytohormone signals

**Figure 6:**
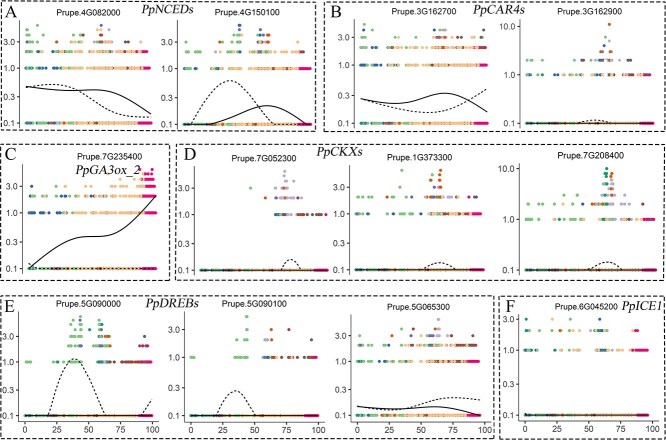
Key genes of reproductive meristem development respond to cold exposure and phytohormone signal pathways**.** Scatter plots based on branched expression analysis modeling of hormone-related genes for ABA biosynthesis (**A**), ABA receptor (**B**), GA biosynthesis (**C**), and cytokinin oxidases (**D**), and abiotic stress genes for cold response genes (**E** and **F**). Colored dots indicate cells from different clusters; the *X*-axis represents the pseudotime differentiation trajectory of flower buds and the *Y*-axis shows expression levels.

Besides the genes of the MADS-box family, environmental cues and phytohormone signals also play crucial roles in reproductive meristem development. Two ABA biosynthetic genes (*Prupe.4G082000* and *Prupe.4G150100*) primarily expressed in vegetative organs and reproductive tissues except the style showed a decreasing trend (Fig. 6A). The former was also shown to be regulated by *PpDAM6* in our previous study [46]. A similar expression profile was observed for *PpCAR4-1* and *PpCAR4-2*, which encode an ABA receptor C2-domain-containing protein family (Fig. 6B). A *PpGA3ox-2* (*Prupe.7G235400*) gene preferentially expressed in anther, showed a sustained high expression trend suggesting the content of GA was increased alongside dormancy release (Fig. 6C). In addition to ABA and GA, three cytokinin oxidases (CKX1, CKX3, and CKX5), responsible for CK degradation, showed decreased expression during chilling accumulation (Fig. 6D, Supplementary Data Table S8).

Cold acclimation is essential to ensure the development of peach flower organs. Many genes of cold-response pathways are differentially expressed at the end of the cold accumulation period [44]. We identified a DREB1A/CBF (dehydration responsive element-binding factor 1A/C-repeat-binding factor) gene, *Prupe.5G090100*, and a DREB1B/CBF gene, *Prupe.5G090000*, with a similar expression pattern; the former has higher accumulation in the style. This demonstrates that they may play different roles in flower bud development (Fig. 6E). What needs illustration is that the latter transcript had a relatively low expression level during the whole dormancy process. The CBF/DREB3D transcript (*Prupe.5G065300*) was upregulated in the initial phase and progressively decreased its expression towards chilling accumulation. Additionally, the expression of ICE1, also named SCREAM (SCRM1), remained unchanged during the cold accumulation process (Fig. 6F, Supplementary Data Table S8).

### Key genes of anther development provide a key reference for activity–dormancy

The essence of the activity–dormancy transition for flower buds is closely related to the levels of anther development. To identify the key developmental stages of pollen maturation under a stressful environment, we performed trajectory analysis of all three samples. Ad1 and Ad2 had a very close differentiation distance, and Ad3 had distinct differentiation compared with the first two samples (Supplementary Data Fig. S7A). These results show that the rate of flower bud development was slower in the early stages of dormancy and Ad2 and Ad3 acted as a key transition phase of pollen maturation. Identification of the close connection of all developmental anther subclusters allowed us to construct a developmental trajectory of anther cell differentiation over pseudotime. To further verify the hypothesis, the clusters of anther development 1, 2, and 7 in Ad1, the clusters 1, 2, 5, and 8 in Ad2, and the clusters 1, 2, 5, and 7 in Ad3 were identified for the trajectory analysis. Cells from anther subclusters 1, 2, 5, and 8 of Ad2 were grouped at one end of the branch and cells from subclusters 1, 2, 5, and 7 of Ad3 were grouped at another branch, showing specific expression patterns of anther marker genes at different development phase (Supplementary Data Fig. S7B).

GO and KEGG functional enrichment analyses of anther development genes of Ad2 and Ad3 samples showed that these oxidation–reduction process, plant hormone signal transduction (auxin response), and citrate cycle (fatty−acyl−CoA reductase) pathways are significantly enriched (Supplementary Data Fig. S7C and D). In this transition phase, several genes concerned in auxin biosynthesis, bHLH, and MYB played crucial roles in activity–dormancy transition. The top 10 genes of clusters 1, 2, 5, and 8 in Ad2 and clusters 1, 2, 5, and 7 in Ad3 for pollen development were selected, and gene function annotation indicated that the YUC2 gene (*Prupe.7G231200*) and SAUR-like auxin-responsive protein family (*Prupe.8G081400* and *Prupe.8G081600*) were preferentially expressed in reproductive cells. Additionally, a gene of the pectin methylesterase inhibitor superfamily (*Prupe.8G264000*) was also identified by enrichment analysis (Supplementary Data Fig. S8), which suggests that it is a housekeeping isoform involved in the maintenance of cell wall integrity throughout the dormancy of buds [64].

**Figure 7:**
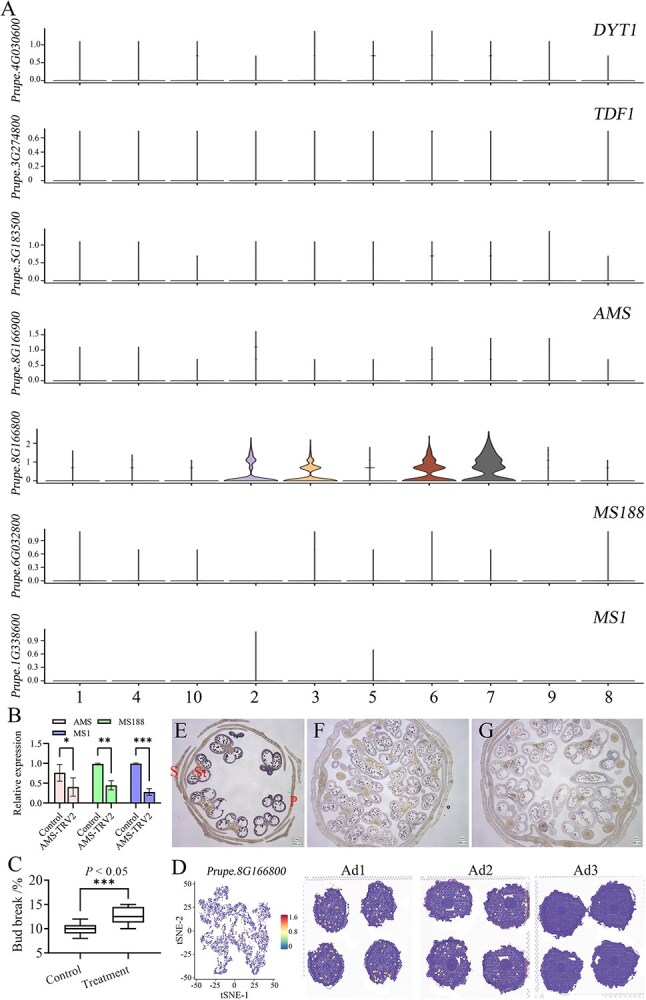
Identification of key genes related to reproductive tissue development was performed alongside the activity–dormancy cycle. **A** Violin plots show expression levels of representative pollen development marker genes. **B** Analysis of the expression trend of these two genes (MS188 and MS1) is displayed in AMS-silenced (AMS-TRV2) lines using peach flower bud. Error bars show the average value ± standard deviation of three independent biological replicates. **C** Bud break was induced earlier in treatment group plants. Percentages of bud break in six plants of control and AMS-VIGS-treated groups are shown. **D** t-SNE graph showing the distribution and expression level of the*Prupe.8G166800* (*AMS*) gene. Dark blue represents low expression levels and red represents high expression levels**. E**–**G**  *In situ* hybridization study of AMS mRNAs during cold accumulation in three peach buds. Images are lateral sections of flower buds labeled by antisense mRNA probes at different dormancy stages. Bluish violet represents the hybridization signal. P, petal; S, sepal; St, stamen. Scale bar, 205 μm.

These genes involved in anther development and meiosis could be used as ideal biomarkers to predict dormancy transition, e.g. a MADS-box gene (*Prupe.5G243900*) for the abundance of Golgi bodies in cluster 6 was identified, suggesting the cluster contained tapetum cells for providing nutrition. The gene *Prupe.1G192200* (*ppa006506m*) is a putative ortholog of *LAP3* [36], which is expressed in clusters 2, 3, 6, 7, and 9 and essential for proper exine formation of pollen [65]. The *Arabidopsis* potential ortholog of *Prupe.2G097200* (*ppa008351m*) (*AtbHLH91*/*At2g31210*) for the pollen maturation pathway belongs to the bHLH-type TF family [36], which was expressed in clusters 2, 6, and 7 and interacted at the protein level with AMS (*Prupe.8G166800* and *Prupe.8G166900*) and DYT1, two bHLH-type factors involved in tapetum development and pollen wall formation [66, 67]. DYT genes functioned during the early stage of tapetum development, which played an important role in cell wall formation. For example, DYT1 (*Prupe.4G030600*) regulates the formation of the pollen wall, primarily via TDFs (*Prupe.3G274800* and *Prupe.5G183500*) [68], and the related genes in the tapetum were downregulated in the dyt1 mutant [66]. MS1, a direct target of MS188, regulates the expression of key sporophytic pollen coat genes [16]. The hypothesized dormancy regulation model of DYT1-TDF1-AMS-MS188 (*Prupe.6G032800*)-MS1 (*Prupe.1G338600*) was displayed in peach (Fig. 7A). We knocked down the expression of AMS by a VIGS (virus-induced gene silencing) experiment; the expression trend of these two genes (MS188 and MS1) in the silenced lines (AMS-TRV2) was consistent with AMS (Fig. 7B), and bud break was induced earlier in six independent AMS-VIGS plants (Fig. 7C). These results show the conservative mechanism of anther development in different species.

Since few marker genes for flower bud development during the dormancy stage were known in peach, ST-seq provides a convenience method for finding key marker genes. To this end, we selected the key DEG in anther and identified it by RNA *in situ* hybridizations. The enrichment of bHLH TF in cluster 2—confirmed by *in situ* hybridization analysis of *AMS* (*Prupe.8G166800*), which encodes the bHLH TF—revealed its specific transcription in anther cells (Fig. 7D); as *Arabidopsis* mutants of bHLH TF exhibited irregular degradation of the tapetum and defects in the pollen wall [55, 69], its expression level showed a decreasing trend with dormancy progression (Fig. 7E–G), suggesting that it might play a crucial role in anther development.

## Discussion

During the development and differentiation of the identical organism, the cells of the root and inflorescence can undergo tremendous changes, which reflects the heterogeneous results of cell differentiation within the same tissue [70]. Ultimately, the key evidence for major cellular components can be gained from heterogeneity in cellular gene expression levels [71]. Therefore, the analysis of specialized cells and their interactions is of great significance for a comprehensive understanding of the function of plant tissues and biological systems (Fig. 7A) [35]. The identification of key genes in different clusters of each sample provides key references for further detailed research on the function of these genes (Fig. 4A). Using the ST-seq atlas of different tissues, we can accurately describe their functions and response to environmental cues and the underlying molecular networks that drive their activities, especially during the dormancy–activity transition of flower buds before anthesis. Moreover, the spatial transcriptome data detect similar amounts of transcribed genes when compared with standard bulk RNA-seq of the peach flower bud in our previous study [60], suggesting the application of this technology would greatly benefit the study of the plant.

In our study, we analyzed spatial transcriptomic datasets to investigate dynamic changes in gene expression functioning along with floral bud development. The samples were distributed through dormancy and dormancy release stages and the tissue morphology of each part was visible. However, the trajectory analysis still showed the development dynamics from the outside to the inside, indicating that the development of flower buds did not pause, even in a dormant state. Floral organ initialization and identity determination were modeled by comparing the spatio-temporal expression distribution of genes and identifying thousands of genes that are preferentially expressed in specific tissues/stages, including the well-known MADS-box genes and many other potential downstream genes (Fig. 5A and B). The essence of dormancy release is internal anther development, and the hidden regulatory mechanism is still worth further exploration (Fig. 7A and B). This study comprised a systematic investigation of flower bud dormancy in peach and provided an important reference for further study of the gene regulatory network behind this complex process.

Nevertheless, because of the presence of cell walls, challenges remain in procedures of sample preparation when applying spatial transcriptome sequencing to plant tissues, and digestive systems suitable for different tissues have yet to be developed. Additionally, plant cells differ widely in shape and size, leading to large variations in the amount of RNA molecules captured through the spots of the same size. At the same time, the spots (55 μm width) can cover a few cells, resulting in reduced resolution when cell typing is performed. Therefore, there is a need for the development of computational algorithms for cell clustering by integrating information on cell size and shape in histological images. Dormancy release and the subsequent flowering are two discrete processes, and the identification of meiosis and anther development genes provides evidence for accurately forecasting dormancy transition [4].

## Materials and methods

### Sample collection

The peach cultivar ‘Zhou Xing Shan Tao’ (*Amygdalus persica* Linn. var. *persica* f. *pyramidalis* Dipp.) (abbreviated Ad) grown in the research orchard of Zhengzhou Fruit Research Institute, Zhengzhou, China, was used in this study. Flower bud samples used for spatial transcriptome sequencing (10x Genomics Visium platform) were collected for three time points (20 December, 18 January, and 15 February corresponding to dormancy, dormancy release, and eco-dormancy, respectively) from 10-year-old trees. The anther color of Ad1 and Ad2 remained roughly similar green; however, the anther color of Ad3 turning yellowish. Specific experimental processes were performed on a 10x Visium platform with a resolution of 55 μm. The flower buds are at the stage of dormancy and eco-dormancy before anthesis. Specifically, the first samples on slide 1 are at anther formation; the second samples on slide 2 are in meiosis; and the third samples on slide 3 contain mature pollen grains. Other peach buds with no scales were fixed in *in situ* hybridization solution or stored in a −80°C freezer for extracting mRNA analysis.

### 10x Visium spatial RNA-seq data preprocessing

The sequencing and data analysis were completed by Shanghai Oebiotech Co., Ltd. Specifically, the Space Ranger (Version 1.2, default parameter) software pipeline provided by 10x Genomics was used to process Visium spatial RNA-seq output and brightfield microscope images to detect tissues and align reads using the STAR [43] aligner, generate feature-spot matrices, then perform clustering and gene expression analysis, and place spots in spatial context on the slide image. The unique molecular identifier (UMI) count matrixes were digested using the R package of Seurat (version 3.2) [72]. The default normalization method for Seurat is LogNormalize. Data standardization is based on sctransform software [73] to account for the difference across the different data points.

### Dimension reduction and cluster analysis

Top variable genes from the different clusters were identified using the previously described method [74]. Cells from the different tissues were clustered using a graph-based clustering approach and were visualized in two dimensions using the UMAP or t-SNE method. The t-SNE method adjusts the soft boundary between global (i.e. inter-subpopulation) and local (i.e. intra-subpopulation) structures by means of the Perplexity parameter. The larger the Perplexity, the more tightly the cells are distributed, and the more weakened is the information about the local structures. UMAP uses two computational methods based on Pearson coefficient similarity and cosine similarity (Seurat default in R package).

### Pseudotime analysis

Pseudotime trajectories of the spatial transcriptome were constructed using variable genes with Monocle 2 with default parameters. This approach works by placing ST-seq data on trajectories that correspond to biological processes such as cell differentiation, using machine learning techniques such as inverse graph embedding to learn trajectories that describe how cells transition from one state to another. The trajectory is constructed using the DDRTree method. Monocle 2 used the BEAM (branched expression analysis modeling) method to analyze the cell data after quasi-time sequencing and the designated nodes, and then found the branch-related differential genes, focusing on the influence of changes in these marker genes.

### Functional enrichment analysis for differentially expressed genes

For difference enrichment analysis between groups, the screening criteria for DEGs were FC > 1.5 and *P* < 0.05, calculated using the FindMarker function. For marker genes in each cluster, the FindAllMarkers function was used for analysis; the screening criteria were logfc.threshold > 0, min.pct (percentage of cells expressing the gene) > 0.25.

### Experiment for *in situ* hybridization and expression analysis

The probe was designed by a double-ended isotope labeling method. At the same time, fresh peach buds were fixed with 4% paraformaldehyde, and the tissue samples after hybridization were cut into 8-μm thick sections on the microtome. The specific operation process can be referred to in a previous study [31]. For qRT–PCR analysis, total RNA was isolated and the analytical method was as previously described [60]. The primers used for *in situ* hybridization are listed in Supplementary Data Table S9.

### Virus-induced gene silencing experiment

This was based on the method described in our previous research [46].

### qRT–PCR and statistical analysis

The qRT–PCR experiment and calculation method were described in our previous studies [60]. Statistical variations were tested by the two-way ANOVA through GraphPad Prism 9.0.0 software (https://www.graphpad.com/scientific-software/prism/).

## Supplementary Material

Web_Material_uhaf028

## Data Availability

All relevant data are available within the article and the supplementary data.

## References

[1] Jean-Michel L, Yann G, Gustavo M. et al. Differentiated responses of apple tree floral phenology to global warming in contrasting climatic regions. *Front Plant Sci*. 2015;6:1054–66 26697028 10.3389/fpls.2015.01054 PMC4678210

[2] Shi T, Luo W, Li H. et al. Association between blooming time and climatic adaptation in *Prunus mume*. *Ecol Evol*. 2020;10:292–306 31988729 10.1002/ece3.5894 PMC6972806

[3] Atkinson CJ, Brennan RM, Jones HG. Declining chilling and its impact on temperate perennial crops. *Environ Exp Bot*. 2013;91:48–62

[4] Singh RK, Svystun T, Aldahmash B. et al. Photoperiod- and temperature-mediated control of phenology in trees – a molecular perspective. *New Phytol*. 2017;213:511–24 27901272 10.1111/nph.14346

[5] Knight TA . Account of some experiments on the ascent of the sap in trees. *Philos Trans R Soc Lond*. 1801;91:333–53

[6] Luedeling E, Girvetz EH, Semenov MA. et al. Climate change affects winter chill for temperate fruit and nut trees. *PLoS One*. 2011;6:e20155 21629649 10.1371/journal.pone.0020155 PMC3101230

[7] Richardson EA . A model for estimating the completion of rest for 'Redhaven' and 'Elberta' peach trees. *HortScience*. 1974;9:331–2

[8] Ruiz D, Campoy JA, Egea J. Chilling and heat requirements of apricot cultivars for flowering. *Environ Exp Bot*. 2007;61:254–63

[9] Weinberger J. Chilling Requirements of Peach Varieties. Proceedings of American Society for Horticultural Science. 1950;56:122–8

[10] Fadón E, Herrera S, Herrero M. et al. Male meiosis in sweet cherry is constrained by the chilling and forcing phases of dormancy. *Tree Physiol*. 2021;41:619–30 32453409 10.1093/treephys/tpaa063

[11] Herrera S, Lora J, Fadón E. et al. Male meiosis as a biomarker for endo- to ecodormancy transition in apricot. *Front*. *Plant Sci*. 2022;13:842333 10.3389/fpls.2022.842333 PMC9021868 35463418

[12] Julian C, Herrero M, Rodrigo J. Anther meiosis time is related to winter cold temperatures in apricot (*Prunus armeniaca* L.). *Environ Exp Bot*. 2014;100:20–5

[13] Yao X, Tian L, Yang J. et al. Auxin production in diploid microsporocytes is necessary and sufficient for early stages of pollen development. *PLoS Genet*. 2018;14:e1007397 29813066 10.1371/journal.pgen.1007397 PMC5993292

[14] Wang K, Guo ZL, Zhou WT. et al. The regulation of sporopollenin biosynthesis genes for rapid pollen wall formation. *Plant Physiol*. 2018;178:283–94 30018171 10.1104/pp.18.00219 PMC6130021

[15] Zhu J, Lou Y, Xu X. et al. A genetic pathway for tapetum development and function in *Arabidopsis*. *J Integr Plant Biol*. 2011;53:892–900 21957980 10.1111/j.1744-7909.2011.01078.x

[16] Lu JY, Xiong SX, Yin W. et al. MS1, a direct target of MS188, regulates the expression of key sporophytic pollen coat protein genes in *Arabidopsis*. *J Exp Bot*. 2020;71:4877–89 32374882 10.1093/jxb/eraa219 PMC7410184

[17] Mirgorodskaya OE, Koteyeva NK, Volchanskaya AV. et al. Pollen development in rhododendron in relation to winter dormancy and bloom time. *Protoplasma*. 2015;252:1313–23 25643916 10.1007/s00709-015-0764-y

[18] Cooke JE, Eriksson ME, Junttila O. The dynamic nature of bud dormancy in trees: environmental control and molecular mechanisms. *Plant Cell Environ*. 2012;35:1707–28 22670814 10.1111/j.1365-3040.2012.02552.x

[19] Ariizumi T, Toriyama K. Genetic regulation of sporopollenin synthesis and pollen exine development. *Annu Rev Plant Biol*. 2011;62:437–60 21275644 10.1146/annurev-arplant-042809-112312

[20] Leida C, Conesa A, Llácer G. et al. Histone modifications and expression of DAM6 gene in peach are modulated during bud dormancy release in a cultivar-dependent manner. *New Phytol*. 2012;193:67–80 21899556 10.1111/j.1469-8137.2011.03863.x

[21] Wu F, Shi X, Lin X. et al. The ABCs of flower development: mutational analysis of AP1/FUL-like genes in rice provides evidence for a homeotic (A)-function in grasses. *Plant J*. 2017;89:310–24 27689766 10.1111/tpj.13386

[22] Yamane H, Tomomi O, Hiroaki J. et al. Expressional regulation of *PpDAM5* and *PpDAM6*, peach (*Prunus persica*) dormancy-associated MADS-box genes, by low temperature and dormancy-breaking reagent treatment. *J Exp Bot*. 2011;62:3481–8 21378115 10.1093/jxb/err028 PMC3130173

[23] Bielenberg DG, Wang YE, Li Z. et al. Sequencing and annotation of the evergrowing locus in peach [*Prunus persica* (L.) Batsch] reveals a cluster of six MADS-box transcription factors as candidate genes for regulation of terminal bud formation. *Tree Genet Genomes*. 2008;4:495–507

[24] Costanzo E, Trehin C, Vandenbussche M. The role of WOX genes in flower development. *Ann Bot*. 2014;114:1545–53 24973416 10.1093/aob/mcu123 PMC4204783

[25] Tylewicz S, Petterle A, Marttila S. et al. Photoperiodic control of seasonal growth is mediated by ABA acting on cell-cell communication. *Science*. 2018;360:212–5 29519919 10.1126/science.aan8576

[26] Zheng C, Halaly T, Acheampong AK. et al. Abscisic acid (ABA) regulates grape bud dormancy, and dormancy release stimuli may act through modification of ABA metabolism. *J Exp Bot*. 2015;66:1527–42 25560179 10.1093/jxb/eru519 PMC4339608

[27] Rinne PL, Paul LK, Vahala J. et al. Axillary buds are dwarfed shoots that tightly regulate GA pathway and GA-inducible 1,3-β-glucanase genes during branching in hybrid aspen. *J Exp Bot*. 2016;67:5975–91 27697786 10.1093/jxb/erw352 PMC5100014

[28] Zheng C, KwameAcheampong A, Shi Z. et al. Distinct gibberellin functions during and after grapevine bud dormancy release. J Exp Bot. 2018;69:1635–48 29385616 10.1093/jxb/ery022 PMC5888973

[29] Ryu KH, Huang L, Kang HM. et al. Single-cell RNA sequencing resolves molecular relationships among individual plant cells. *Plant Physiol*. 2019;179:1444–56 30718350 10.1104/pp.18.01482 PMC6446759

[30] Liu Q, Liang Z, Feng D. et al. Transcriptional landscape of rice roots at the single-cell resolution. *Mol Plant*. 2021;14:384–94 33352304 10.1016/j.molp.2020.12.014

[31] Zong J, Wang L, Zhu L. et al. A rice single cell transcriptomic atlas defines the developmental trajectories of rice floret and inflorescence meristems. *New Phytol*. 2022;234:494–512 35118670 10.1111/nph.18008

[32] Nelms B, Walbot V. Defining the developmental program leading to meiosis in maize. *Science*. 2019;364:52–6 30948545 10.1126/science.aav6428

[33] Omary M, Gil-Yarom N, Yahav C. et al. A conserved superlocus regulates above- and belowground root initiation. *Science*. 2022;375:eabf4368 35239373 10.1126/science.abf4368

[34] Li H, Dai X, Huang X. et al. Single-cell RNA sequencing reveals a high-resolution cell atlas of xylem in *Populus*. *J Integr Plant Biol*. 2021;63:1906–21 34347368 10.1111/jipb.13159

[35] Turco GM, Rodriguez-Medina J, Siebert S. et al. Molecular mechanisms driving switch behavior in xylem cell differentiation. *Cell Rep*. 2019;28:342–351.e4 31291572 10.1016/j.celrep.2019.06.041

[36] Ríos G, Tadeo FR, Leida C. et al. Prediction of components of the sporopollenin synthesis pathway in peach by genomic and expression analyses. *BMC Genomics*. 2013;14:40 23331975 10.1186/1471-2164-14-40 PMC3556096

[37] Trapnell C, Cacchiarelli D, Grimsby J. et al. The dynamics and regulators of cell fate decisions are revealed by pseudotemporal ordering of single cells. *Nat Biotechnol*. 2014;32:381–6 24658644 10.1038/nbt.2859 PMC4122333

[38] Gómez JF, Talle B, Wilson ZA. Anther and pollen development: a conserved developmental pathway. *J Integr Plant Biol*. 2015;57:876–91 26310290 10.1111/jipb.12425 PMC4794635

[39] Canton M, Forestan C, Marconi G. et al. Evidence of chromatin and transcriptional dynamics for cold development in peach flower bud. *New Phytol*. 2022;236:974–88 35860865 10.1111/nph.18393 PMC9804738

[40] Saito T, Bai S, Imai T. et al. Histone modification and signalling cascade of the dormancy-associated MADS-box gene, *PpMADS13-1*, in Japanese pear (*Pyrus pyrifolia*) during endodormancy. *Plant Cell Environ*. 2015;38:1157–66 25311427 10.1111/pce.12469

[41] Yamane H, Takeuchi T, Matsushita M. et al. Expression analysis of apple DORMANCY-ASSOCIATED MADS-box genes in ‘Fuji’ dormant flower buds during flower bud development. *Acta Hortic*. 2019;1261:143–1148

[42] Bačovský V, Čegan R, Tihlaříková E. et al. Chemical genetics in *Silene latifolia* elucidate regulatory pathways involved in gynoecium development. *J Exp Bot*. 2022;73:2354–68 35045170 10.1093/jxb/erab538

[43] Dobin A, Davis CA, Schlesinger F. et al. STAR: ultrafast universal RNA-seq aligner. *Bioinformatics*. 2013;29:15–21 23104886 10.1093/bioinformatics/bts635 PMC3530905

[44] Artlip T, Mcdermaid A, Ma Q. et al. Differential gene expression in non-transgenic and transgenic "M.26" apple overexpressing a peach CBF gene during the transition from eco-dormancy to bud break. *Hortic Res*. 2019;11:86 10.1038/s41438-019-0168-9 PMC6804898 31666956

[45] Cai Y, Wang L, Ogutu CO. et al. The MADS-box gene PpPI is a key regulator of the double-flower trait in peach. *Physiol Plant*. 2021;173:2119–29 34537956 10.1111/ppl.13561

[46] Zhao YL, Li Y, Cao K. et al. MADS-box protein PpDAM6 regulates chilling requirement-mediated dormancy and bud break in peach. *Plant Physiol*. 2023;193:448–65 37217835 10.1093/plphys/kiad291 PMC10469376

[47] Li S, Wuyun TN, Wang L. et al. Genome-wide and functional analysis of late embryogenesis abundant (LEA) genes during dormancy and sprouting periods of kernel consumption apricots (*P. armeniaca* L. × *P. sibirica* L.). *Int J Biol Macromol*. 2024;279:133245 38977045 10.1016/j.ijbiomac.2024.133245

[48] Qian M, Xu L, Tang C. et al. PbrPOE21 inhibits pear pollen tube growth in vitro by altering apical reactive oxygen species content. *Planta*. 2020;252:43 32870426 10.1007/s00425-020-03446-7

[49] Pereira-Santana A, Alcaraz LD, Castaño E. et al. Comparative genomics of NAC transcriptional factors in angiosperms: implications for the adaptation and diversification of flowering plants. *PLoS One*. 2015;10:e0141866 26569117 10.1371/journal.pone.0141866 PMC4646352

[50] Unte US, Sorensen AM, Pesaresi P. et al. SPL8, an SBP-box gene that affects pollen sac development in *Arabidopsis*. *Plant Cell*. 2003;15:1009–19 12671094 10.1105/tpc.010678 PMC152345

[51] Sharma KD, Nayyar H. Regulatory networks in pollen development under cold stress. *Front Plant Sci*. 2016;7:402 27066044 10.3389/fpls.2016.00402 PMC4814731

[52] He SL, Hsieh HL, Jauh GY. SMALL AUXIN UP RNA62/75 are required for the translation of transcripts essential for pollen tube growth. *Plant Physiol*. 2018;178:626–40 30093526 10.1104/pp.18.00257 PMC6181030

[53] Wang P, Lu S, Xie M. et al. Identification and expression analysis of the small auxin-up RNA (SAUR) gene family in apple by inducing of auxin. *Gene*. 2020;750:144725 32360839 10.1016/j.gene.2020.144725

[54] Sun X, Wang Y, Sui N. Transcriptional regulation of bHLH during plant response to stress. *Biochem Biophys Res Commun*. 2018;503:397–401 30057319 10.1016/j.bbrc.2018.07.123

[55] Zhang W, Sun Y, Timofejeva L. et al. Regulation of *Arabidopsis* tapetum development and function by DYSFUNCTIONAL TAPETUM1 (DYT1) encoding a putative bHLH transcription factor. *Development*. 2006;133:3085–95 16831835 10.1242/dev.02463

[56] Zhang W . A GDSL lipase is required for anther and pollen development. *Plant Physiol*. 2020;182:1810–1 32253333 10.1104/pp.20.00278 PMC7140955

[57] Meng X, Zhang Y, Wang N. et al. *Prunus persica* terpene synthase PpTPS1 interacts with PpABI5 to enhance salt resistance in transgenic tomatoes. *Front*. *Plant Sci*. 2022;13:807342 10.3389/fpls.2022.807342 PMC8905318 35283925

[58] Routray P, Li T, Yamasaki A. et al. Nodulin intrinsic protein 7;1 is a tapetal boric acid channel involved in pollen cell wall formation. *Plant Physiol*. 2018;178:1269–83 30266747 10.1104/pp.18.00604 PMC6236609

[59] Yue YZ, Sun J, Huang X. et al. Characterization of a novel anther-specific gene encoding a leucine-rich repeat protein in petunia. *Genet Mol Res*. 2014;13:9889–98 25501199 10.4238/2014.November.27.17

[60] Zhao Y, Li Y, Cao K. et al. Peculiarity of transcriptional and H3K27me3 dynamics during peach bud dormancy. *Hortic Plant J*. 2024;10:38–50

[61] Zhu H, Chen PY, Zhong S. et al. Thermal-responsive genetic and epigenetic regulation of DAM cluster controlling dormancy and chilling requirement in peach floral buds. *Hortic Res*. 2020;7:114 32821397 10.1038/s41438-020-0336-y PMC7395172

[62] Li Z, Lynn RG, Glenn AA. et al. Dormancy-associated MADS genes from the EVG locus of peach [*Prunus persica* (L.) Batsch] have distinct seasonal and photoperiodic expression patterns. *J Exp Bot*. 2009;60:3521–30 19553369 10.1093/jxb/erp195 PMC2724702

[63] Van Der Linden CG, Vosman B, Smulders MJ. Cloning and characterization of four apple MADS box genes isolated from vegetative tissue. *J Exp Bot*. 2002;53:1025–36 11971914 10.1093/jexbot/53.371.1025

[64] Micheli F, Sundberg B, Goldberg R. et al. Radial distribution pattern of pectin methylesterases across the cambial region of hybrid aspen at activity and dormancy. *Plant Physiol*. 2000;124:191–200 10982434 10.1104/pp.124.1.191 PMC59134

[65] Dobritsa AA, Nishikawa S, Preuss D. et al. LAP3, a novel plant protein required for pollen development, is essential for proper exine formation. *Sex Plant Reprod*. 2009;22:167–77 20033437 10.1007/s00497-009-0101-8

[66] Feng B, Lu D, Ma X. et al. Regulation of the *Arabidopsis* anther transcriptome by DYT1 for pollen development. *Plant J*. 2012;72:612–24 22775442 10.1111/j.1365-313X.2012.05104.x

[67] Xu J, Yang C, Yuan Z. et al. The ABORTED MICROSPORES regulatory network is required for postmeiotic male reproductive development in *Arabidopsis thaliana*. *Plant Cell*. 2010;22:91–107 20118226 10.1105/tpc.109.071803 PMC2828693

[68] Gu JN, Zhu J, Yu Y. et al. DYT1 directly regulates the expression of TDF1 for tapetum development and pollen wall formation in *Arabidopsis*. *Plant J*. 2014;80:1005–13 25284309 10.1111/tpj.12694

[69] Sorensen AM, Kröber S, Unte US. et al. The *Arabidopsis* ABORTED MICROSPORES (AMS) gene encodes a MYC class transcription factor. *Plant J*. 2003;33:413–23 12535353 10.1046/j.1365-313x.2003.01644.x

[70] Sunaga-Franze DY, Muino JM, Braeuning C. et al. Single-nucleus RNA sequencing of plant tissues using a nanowell-based system. *Plant J*. 2021;108:859–69 34390289 10.1111/tpj.15458

[71] Fischer C, Metsger M, Bauch S. et al. Signals trigger state-specific transcriptional programs to support diversity and homeostasis in immune cells. *Sci Signal*. 2019;12:eaao5820 31088978 10.1126/scisignal.aao5820

[72] Stuart T, Butler A, Hoffman P. et al. Comprehensive integration of single-cell data. *Cell*. 2019;177:1888–1902.e21 31178118 10.1016/j.cell.2019.05.031 PMC6687398

[73] Hafemeister C, Satija R. Normalization and variance stabilization of single-cell RNA-seq data using regularized negative binomial regression. *Genome Biol*. 2019;20:296 31870423 10.1186/s13059-019-1874-1 PMC6927181

[74] Macosko EZ, Basu A, Satija R. et al. Highly parallel genome-wide expression profiling of individual cells using nanoliter droplets. *Cell*. 2015;161:1202–14 26000488 10.1016/j.cell.2015.05.002 PMC4481139

