# Supplementary material for: Spatiotemporally transcriptomic analyses of floral buds reveal the high-resolution landscape of flower development and dormancy regulation in peach
Source: Hortic Res. 2025 Feb 11;12(5):uhaf029. doi: 10.1093/hr/uhaf029 (PMC11986580; doi:10.1093/hr/uhaf029)
Supplement: Web_Material_uhaf028 [file web_material_uhaf028.zip › Supplemental Figure.docx]

**Figure legends**


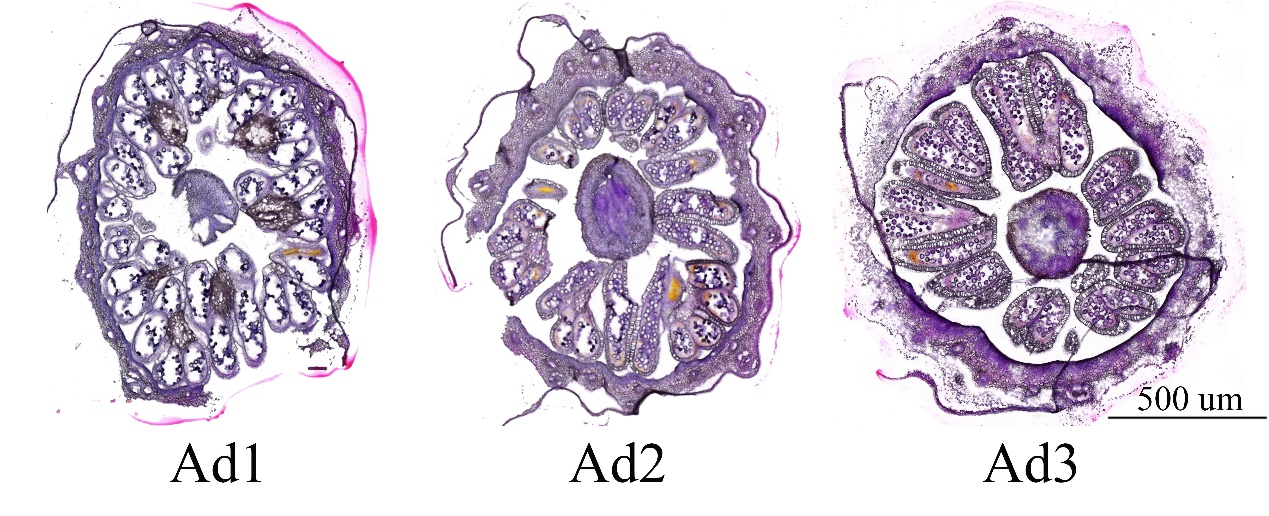


**Supplemental figure S1** The HE (hematoxylin-eosin) staining resules of cross section of three samples (Ad1, Ad2, Ad3), respectively. Scale bars, 500 um.


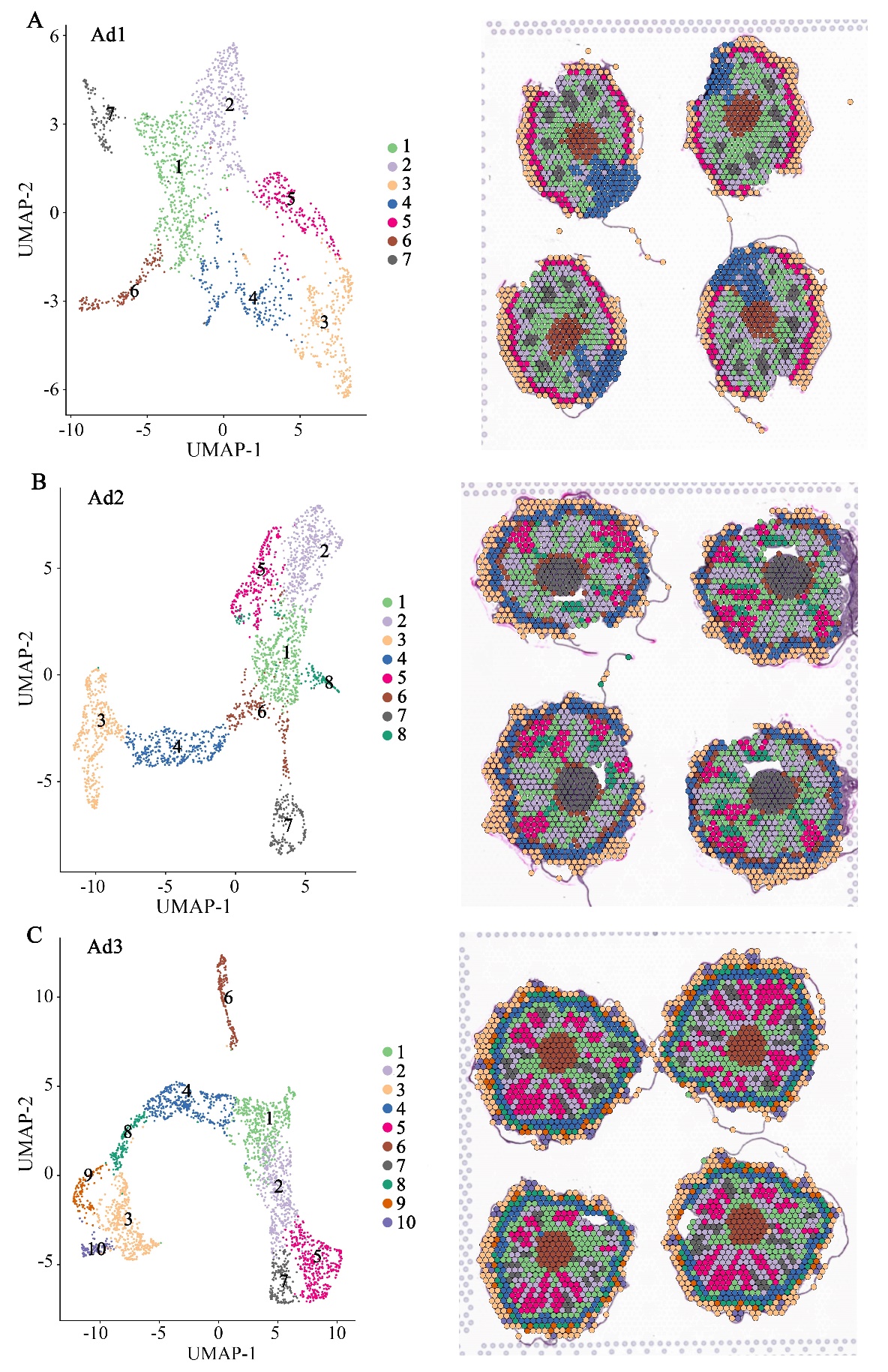


**Supplemental figure S2** The clusters were obtained of three samples by the unsupervised clustering using Seurat and visualized using UMAP algorithms, respectively. Clusters are shown in different colors, and each dot represents a capture area.


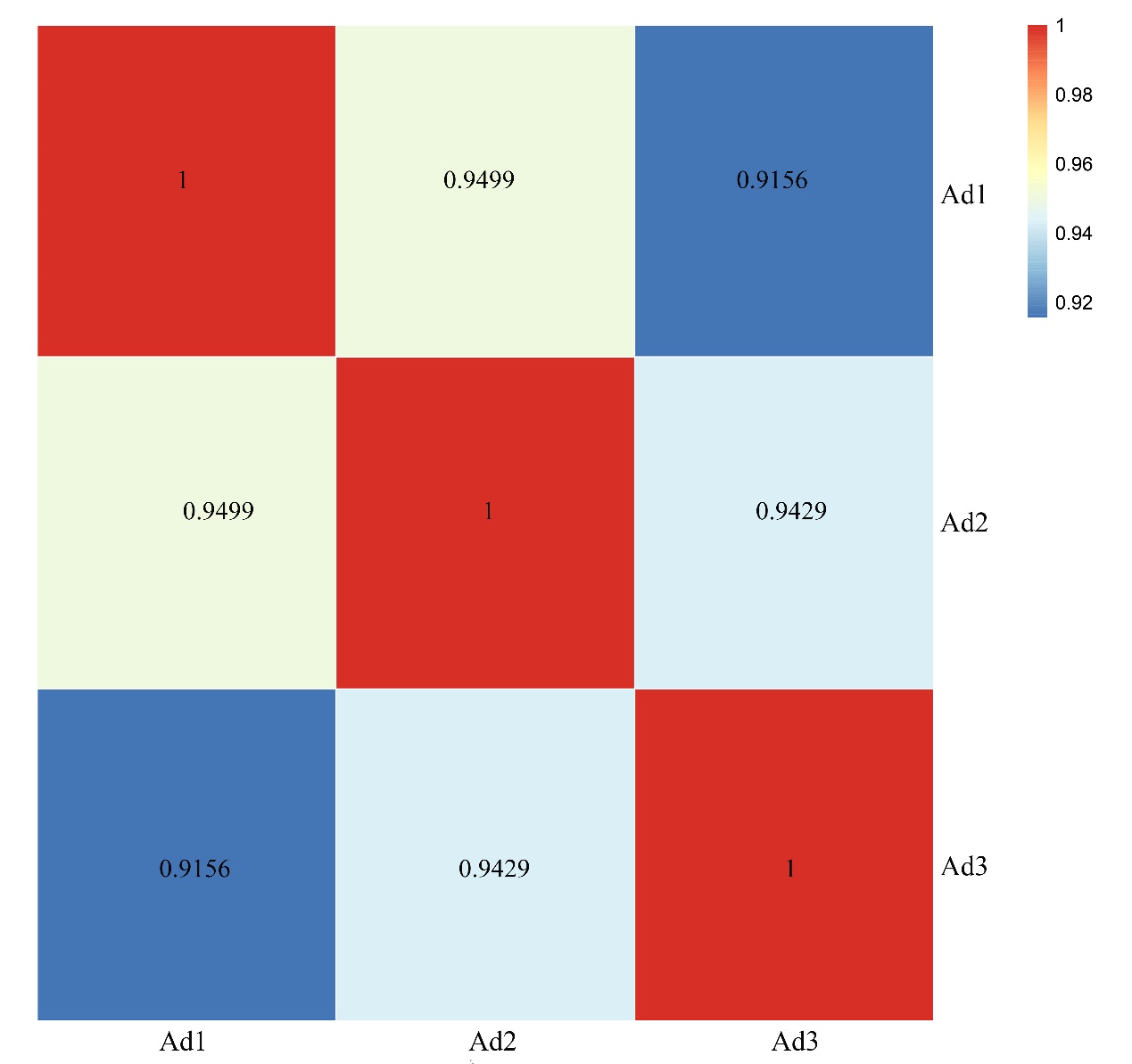


**Supplemental figure S3** The correlation analysis of the three flower bud samples.

**
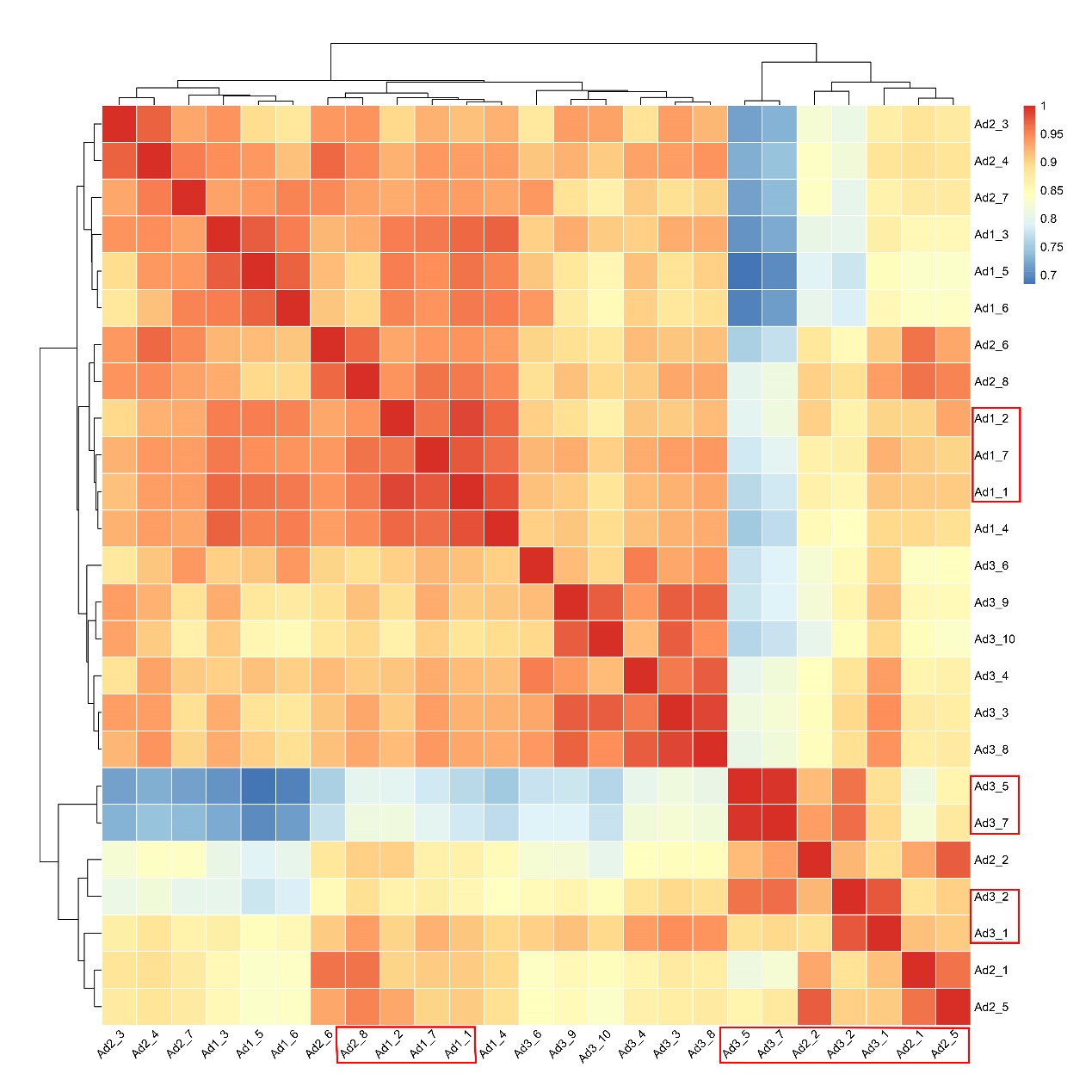
**

**Supplemental figure S4** Cluster analysis based on the whole clusters of the three flower bud samples.

**
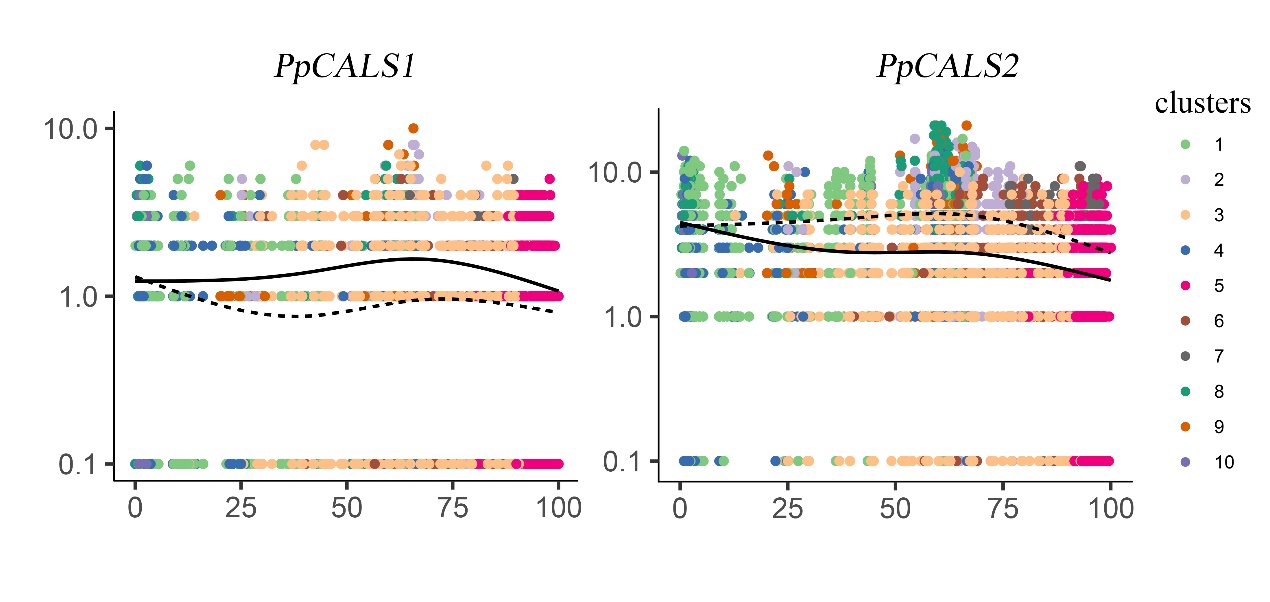
**

**Supplemental figure S5** Scatter plot of callose biosynthesis enzyme genes.

**
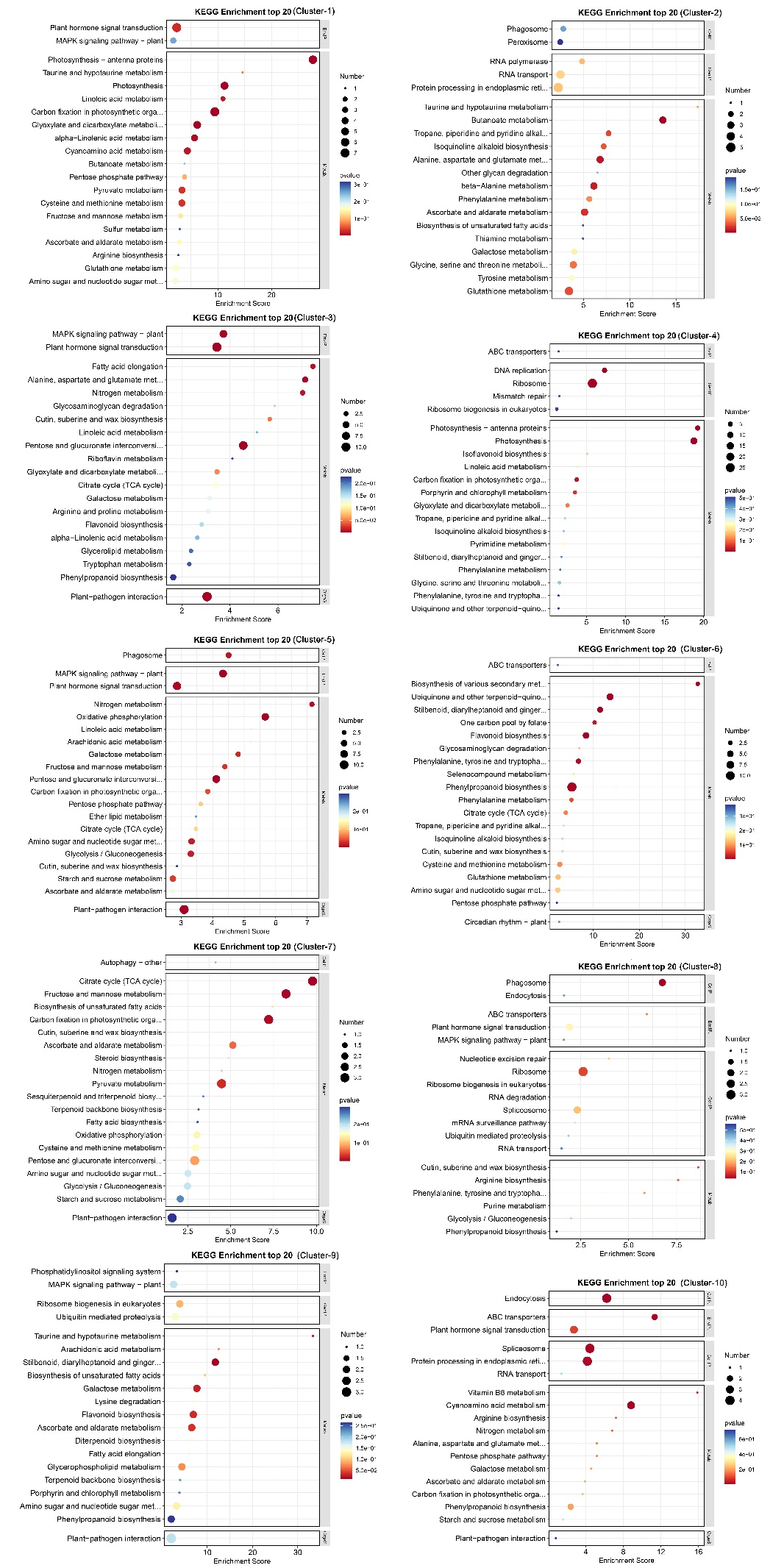
**

**Supplemental figure S6** 10 clusters were analyzed by Kyoto Encyclopedia of Genes and Genomes (KEGG). The highly enriched KEGG terms and pathways are shown in each cluster. The circle size represents enriched gene numbers. Red color and blue color represent significant and insignificant enrichments, respectively, and the degree of significant enrichments on KEGG pathways is shown by *P*-value.

**
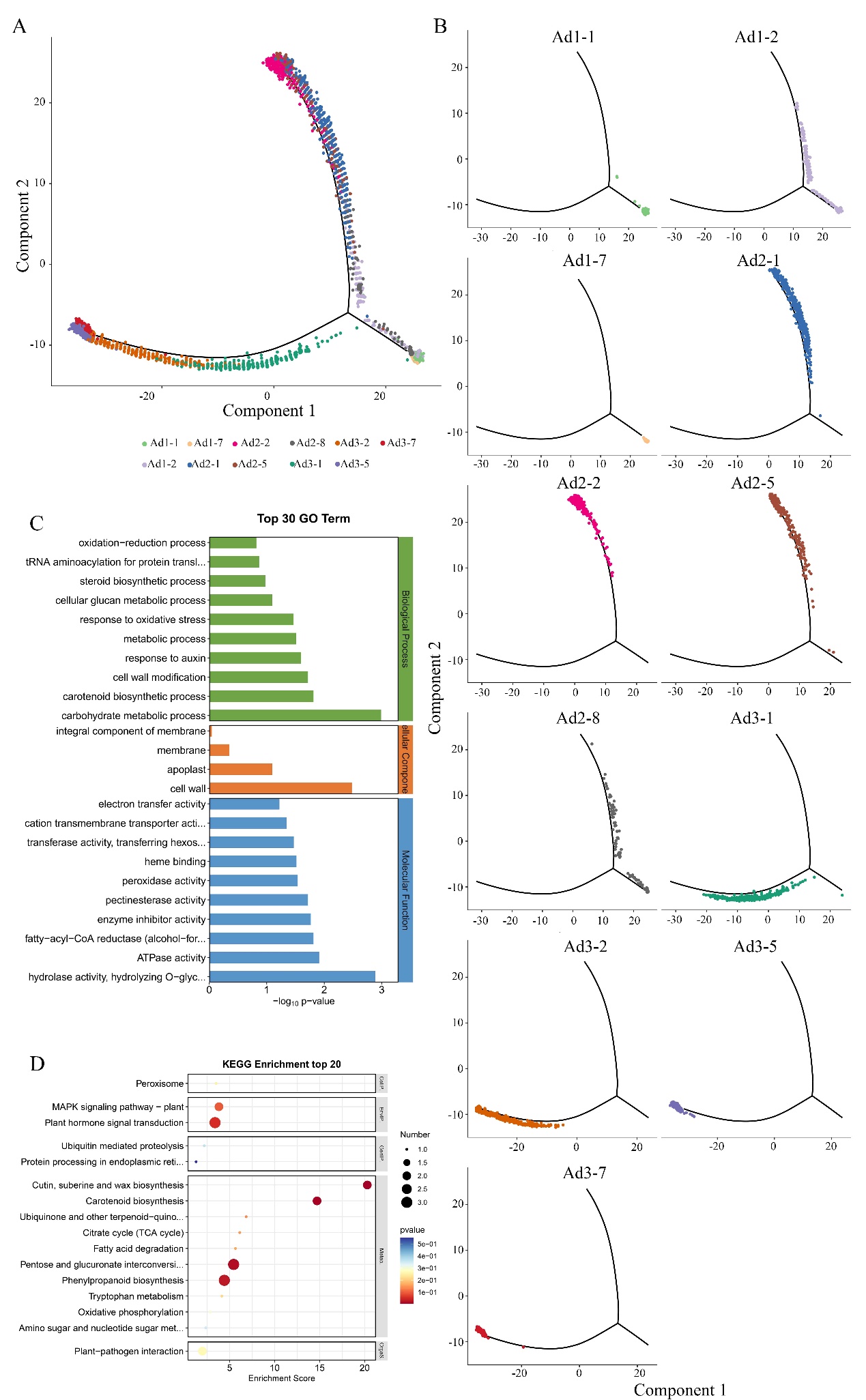
**

**Supplemental figure S7** Functional enrichment and clustering analysis for three samples. (A) Pseudotime trajectory analysis of cluster 1, 2 and 7 in Ad1, cluster 1, 2, 5 and 8 in Ad2, cluster 1, 2, 5 and 7 in Ad3. (B) the detailed clusters of (A). (C and D) The GO and KEGG enrichment analysis were performed using the DEGs of anther development during dormancy transition (*P*< 0.05).

**
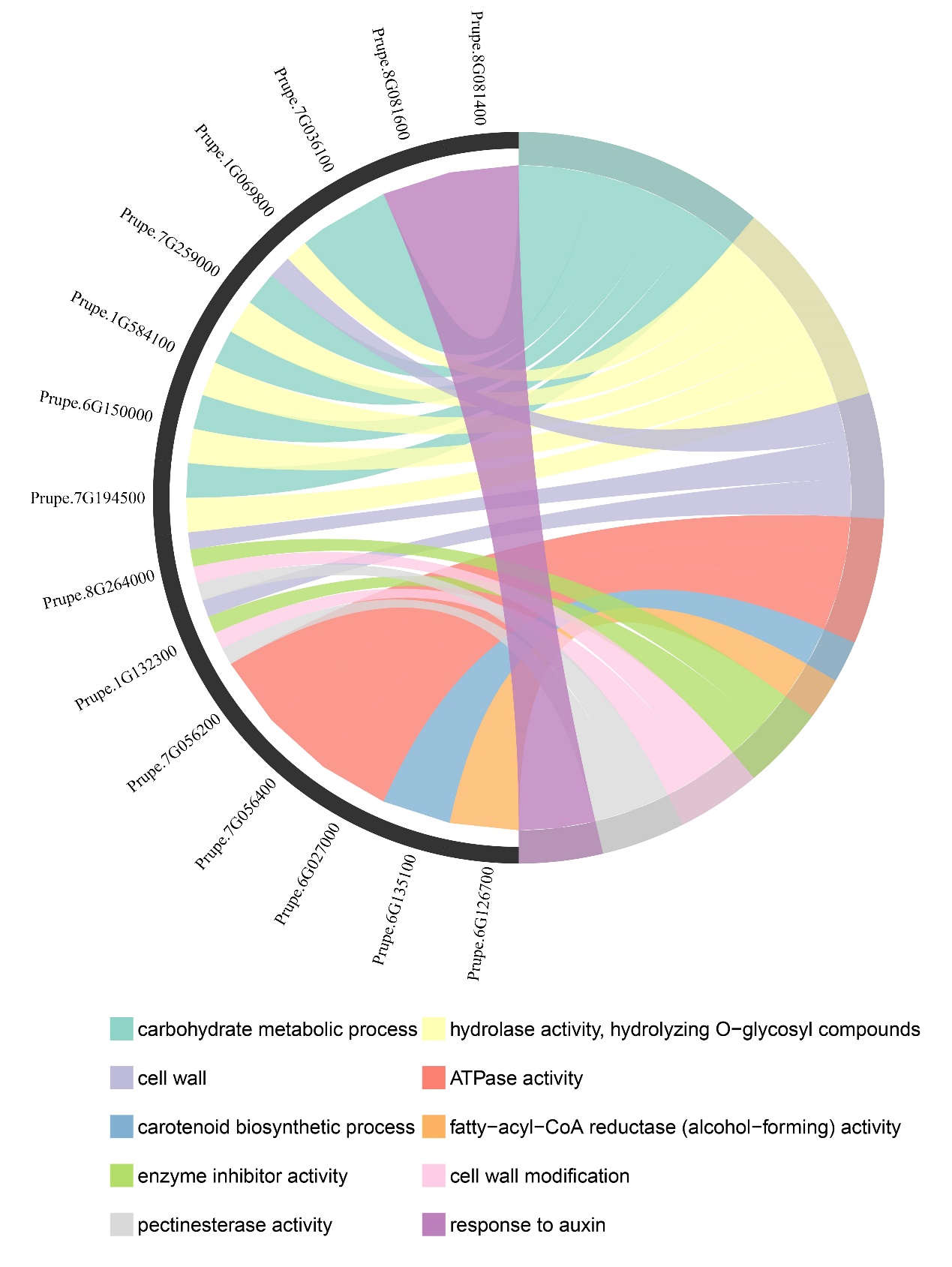
**

**Supplemental figure S8** The identification of key genes using the enrichment analysis of DEGs of anther development.
